# Prevalence of Liver Dysfunction After One-Anastomosis Gastric Bypass: A Systematic Review and Single-Arm Meta-analysis

**DOI:** 10.1007/s11695-025-08219-3

**Published:** 2025-09-09

**Authors:** Andrew Tse, Simeng Li, Jorgen Ferguson, Lee Kyang, Reginald Lord

**Affiliations:** 1 The University of New South Wales, Sydney, Australia; 2 St Vincent’s Hospital Sydney, Darlinghurst, Australia; 3 The University of Notre Dame Australia, Sydney, Australia

**Keywords:** Obesity, One-anastomosis gastric bypass, Complication, Liver function dysfunction

## Abstract

**Background:**

One-anastomosis gastric bypass (OAGB) has gained popularity as a bariatric operation due to its shorter operation time and lower perioperative complication rates, compared with Roux-en-Y gastric bypass (RYGB). However, OAGB is associated with short and long-term complications. Notably, in some reports a subset of patients developed liver dysfunction after OAGB, in some cases causing death or requiring liver transplantation.

**Methods:**

A systematic review and meta-analysis were conducted following PRISMA guidelines. MEDLINE, EMBASE and PubMed databases were searched for studies published from 1946 to June 2024, focusing on the prevalence of liver dysfunction post-OAGB. Data extraction and quality assessment were performed by two independent reviewers. Statistical analysis includes pooled prevalence estimates, subgroup analysis against biliopancreatic limb length and regions of the included studies, sensitivity analysis and public bias assessment by Egger’s test.

**Results:**

Of the 3223 identified articles, 7 studies met the inclusion criteria, involving 2944 patients, with 91 patients developing liver dysfunction post-OAGB. The pooled prevalence of liver dysfunction was 1.2% (95% *CI* 0.3–2.1%), with significant heterogeneity (*I*^2^ = 88.5%, *p* < 0.001). Subgroup analyses did not identify contributors to the heterogeneity. Sensitivity analysis validated the robustness of the findings, and no publication bias was detected by the Egger’s test.

**Conclusion:**

The prevalence of liver dysfunction post OAGB is low but clinically significant, warranting intense postoperative care and regular liver function monitoring. The lack of extensive data on this topic is a limitation, but as the first study to summarise current evidence, this study provides a foundation for future research.

**Supplementary Information:**

The online version contains supplementary material available at 10.1007/s11695-025-08219-3.

## Introduction

Obesity is a global health care issue, for which bariatric surgery is established as an effective treatment that provides substantial weight loss and resolution of obesity associated medical problems [1]. One-anastomosis gastric bypass (OAGB) was first described by Rutledge in 1997 [2] as an alternative to Roux-en Y gastric bypass (RYGB). Although sleeve gastrectomy (SG) and RYGB remain the most performed procedures, the popularity of OAGB has gradually increased worldwide, and it is currently estimated that OAGB accounts for approximately 10% of all bariatric operations globally [3].

OAGB offers advantages such as a shorter operation time and a lower incidence of perioperative complications, compared with RYGB [4]. However, multiple short and long-term complications have been reported, such as bile reflux, marginal ulceration, and malnutrition [1, 5, 6]. An uncommon but serious complication is acute liver dysfunction, which can be fatal. Kruschitz et al. conducted a study comparing liver parameters between OAGB and RYGB in 50 patients, revealing significantly deteriorating liver function in OAGB patients within the first-year post-surgery [7]. Similarly, Eilenberg et al. also published a case series involving 5 OAGB patients developing severe liver dysfunction [8]. One of the five patients eventually progressed to decompensated liver failure requiring a liver transplantation [8]. These findings emphasized the need for more intensive postoperative care and closed liver function monitoring in patients undergoing OAGB. The low rate of complications of OAGB is the reason it was approved by the International Federation for the Surgery of Obesity and Metabolic disorders (IFSO) as a mainstream bariatric procedure in 2017 [9]; acceptance by the American Society for Metabolic Bariatric Surgery was not until May 2022. The potential for severe and even fatal liver dysfunction after OAGB prompted this study to investigate the prevalence and clinical presentation of this complication. A thorough literature search was conducted when preparing this meta-analysis; no systematic review or meta-analysis has previously reported the collective prevalence of liver dysfunction post OAGB.

## Methods

This systematic review and meta-analysis were conducted according to the Preferred Reporting Items for Systematic Reviews and Meta-analyses (PRISMA) guidelines [10]. This review had been registered with PROSPERO (number provided in cover letter). We systematically searched PubMed, Embase and Medline for available studies published from 1946 to June 27, 2024. The search strategy was developed using the concepts of “one anastomosis gastric bypass” OR “mini-gastric bypass” OR “omega loop gastric bypass” OR “liver function test.” We limited to English language, full-text and human studies. The detailed search strategy is provided in Supplementary File: Table S1. We review included cohort studies, case series and case reports examining relevant outcomes. As this study is based on previously published data, formal ethics approval was not required. The authors declare no conflicts of interest.

The definition of liver dysfunction varies across studies. Abnormal liver function is defined as liver function test (LFT) values reaching at least twice the upper limit of the normal range. To account for acute changes due to liver retraction, LFTs need to be measured at least 2 weeks after operation.

### Inclusion and Exclusion Criteria

Studies were included if they meet the following criteria: (1) adults (≥ 18 years) who have undergone one-anastomosis gastric bypass surgery, (2) single-arm or multi-arm studies, (3) reported the prevalence of liver dysfunction/failure after OAGB, (4) studies involving patients with a positive medical history for liver dysfunction/failure, (5) written in English, (6) full-text available (7). If multiple articles were published based on the same dataset, only the original study was included. Studies that did not meet the inclusion criteria were excluded.

### Study Selection and Data Extraction

The study selection followed the PRISMA strategy [10]. All citations were downloaded and managed using EndNote 21 software (Clarivate Analytics, Philadelphia, USA). Duplications were removed. Records were screened by 2 independent reviewers (SL and AT). Title and abstracts were reviewed against inclusion criteria and exclusion criteria. Full texts of the selected studies were retrieved for a second round of eligibility screening.

Additionally, references for articles selected were also reviewed for relevant studies not captured by the initial search. Two reviewers (SL and AT) independently extracted data. The variables extracted from each study included study characteristics (publication year, country, sample size and prevalence of liver dysfunction after OAGB), patient demographics (age, sex and initial *BMI* before surgery), preoperative characteristics (liver function tests and liver biopsy), postoperative characteristics (biliopancreatic limb length, postoperative *BMI*, percentage of excess weight loss at 1 year after surgery, postoperative liver function tests, liver biopsy and onset of liver dysfunction). All data were extracted using a standardised extraction form.

### Methodological Quality Assessment

Three independent reviewers (SL, AT and LK) evaluated the quality of included studies [11]. We assessed the potential for bias in included studies using the Joanna Briggs Institute checklists for case reports and cohort studies [12, 13] and provided details of this assessment in tabulated form.

### Statistical Analysis

The statistical software “STATA 14.0” (Stata Corporation, College Station, TX, USA) was used to conduct all statistical analyses in this meta-analysis. The primary measure of effect in this meta-analysis was prevalence, defined as the ratio of cases to the total population [14]. Subgroup analyses were conducted to examine considered factors potentially associated with the prevalence of liver dysfunction following OAGB. The subgroup variables included biliopancreatic limb length (150–200 cm vs. > 200 cm), region (Europe, Asia, Africa, North America and developing country, developed country).

Heterogeneity among the studies was assessed using the *I*^2^ statistic [15]. When *I*^2^ value was > 50%, a study was considered to have high heterogeneity and a random-effects analysis was used to pool these results. When *I*^2^ value was < 50%, fixed-effect model was used. Statistical tests used 2-tailed *P* value of < 0.05 for significance. Sensitivity analysis was used to determine the effect of individual studies on overall prevalence estimates by sequentially excluding each study [16]. Publication bias was assessed by visual inspection of the funnel plot and Egger’s test [17, 18]. In Egger’s test, *p* < 0.05 suggested publication bias.

## Results

### Study Characteristics

**Fig. 1:**
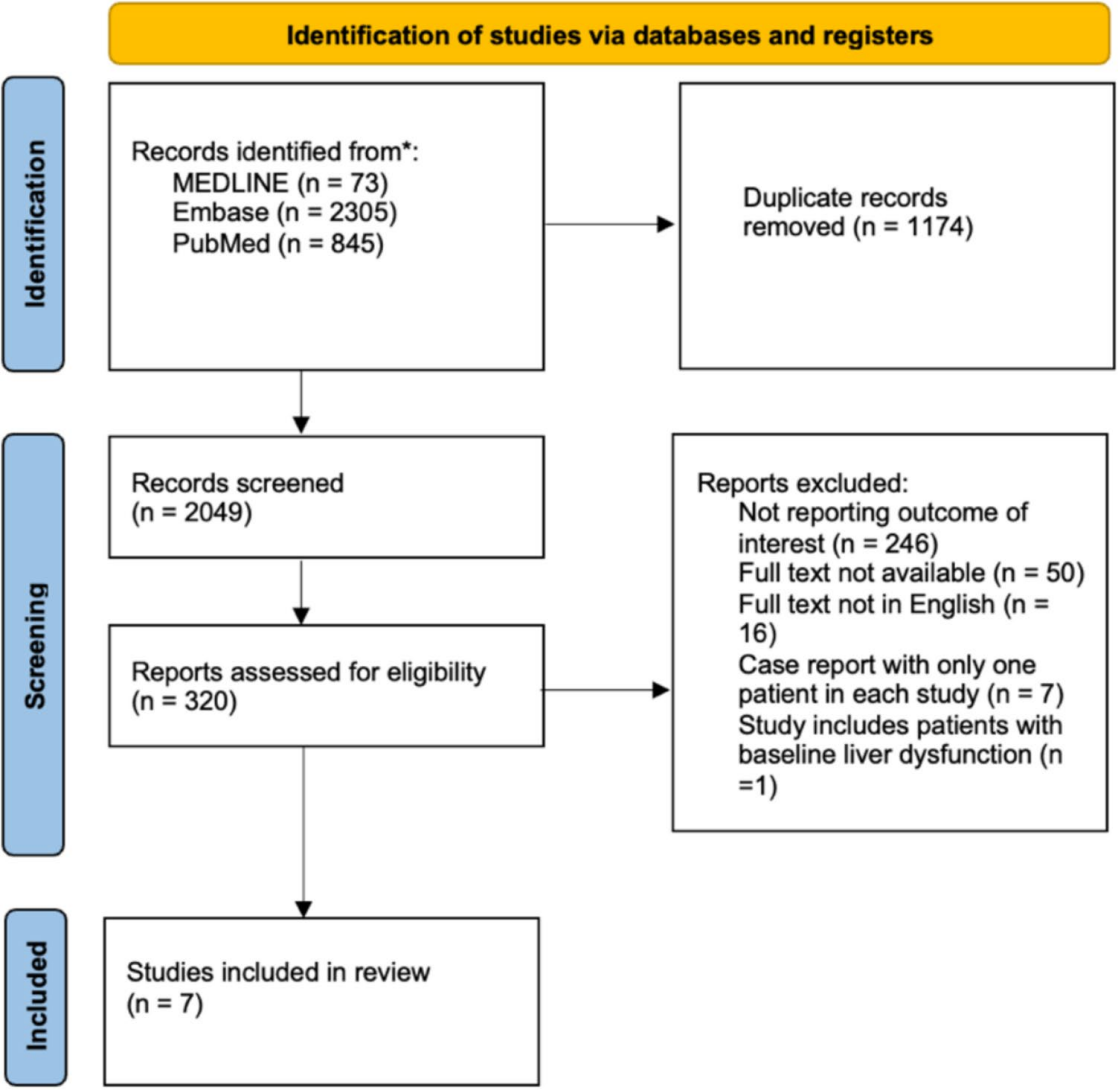
PRISMA flowchart

A total of 3223 articles were identified through database searches. After removing 1174 duplicates, 2049 articles remained. A total of 1729 studies were excluded after title and abstract screening. Three hundred twenty articles were retrieved for full-text screening. Three hundred thirteen articles were excluded due to the lack of full text, non-English language or unavailability through the search databases. Fifteen studies reported prevalence of liver dysfunction following OAGB [8, 19–30]. The quality of included studies was assessed and presented in Tables S2 and S3. The patient preoperative and postoperative characteristics from these studies will still be discussed in this study. Of the 15 studies, eight were excluded because they were case reports, each involving only a single patient [8, 19, 21, 28, 29, 31–33]. As a result, seven studies were included in the meta-analysis [7, 8, 19–21, 23, 26–34] (Fig. 1). Of the included studies, two studies were conducted in UK [19, 20], with four in Iran [21, 26, 29, 31], one in Saudi Arabia [34], one in Egypt [23], one in Mexico [32], one in Austria [7], one in India [27], one in Jordan [28], one in Israel [30] and one in Netherlands [33]. Six were retrospective cohort studies [7, 19, 20, 23, 27, 30]. Detailed characteristics of included studies are presented in Table 1.

**Table 1:** All the studies included in this review with sample size, prevalence of liver dysfunction after OAGB

Included study	Type of study	Country of study	Sample size (*N*)	Prevalence of liver dysfunction (*n*)	BPL length < 150 cm	PBL length 150–200 cm	PBL length > 200 cm
Hussain et al. (2019)	Retrospective	UK	925	2	386	539	0
Motamedi et al. (2017)	Case report	Iran	1	1	0	1	0
Al-Garzaie et al. (2022)	Case series	Saudi Arabia	5	1	0	5	0
Elgeidie et al. (2020)	Retrospective	Egypt	692	2	0	692	0
Sotelo et al. (2023)	Case report	Mexico	1	1	0	1	0
Kruschitz et al. (2016)	Retrospective	Austria	25	23	0	25	0
Hussain et al. (2018)	Retrospective	UK	527	2	0	527	0
Khalaj et al. (2019)	Case series	Iran, Canada	189	2	0	189	0
Ahuja et al. (2018)	Retrospective	India	101	1	0	81	20
Haddad et al. (2020)	Case report	Jordan	1	1	0	0	1
Eilenberg et al. (2017)	Case series	Austria	5	5	1	2	2
Kermansaravia et al. (2016)	Case report	Iran	1	1	0	0	1
Motamedi et al. (2017)	Case report	Iran	1	1	0	1	0
Spivak et al. (2017)	Registry-based cohort study	Israel	469	47	0	469	0
Van Golen et al. (2022)	Case series	Netherlands	1	1	0	1	0

### Patient Characteristics

The 15 studies involved a total of 2944 individuals, of which 91 developed liver dysfunction following OAGB. This cohort had a median age of 43.9 years (range 29–62 years) and a female/male ratio of 8. The median body mass index (*BMI*) of the patients at the time of surgery was 47.1 kg/m^2^ (range 41.3–63.7 kg/m^2^). Preoperative characteristics of patients developed liver dysfunction following OAGB and are presented in Table 2. One patient (1%) had OAGB with a biliopancreatic limb (BPL) length < 150 cm. Eighty-two patients (90%) had OAGB with a BPL length 150–200 cm; 8 patients (9%) had OAGB with a PBL length > 200 cm (Table S4). The median *BMI* of patients after surgery was 25 kg/m^2^ (range 15.11–42 kg/m^2^). All 91 patients developed signs or features of liver dysfunction at a median of 12 months after OAGB (range 3–36 months). Postoperative characteristics of patients developed liver dysfunction following OAGB and are presented in Table 3.

**Table 2:** Preoperative characteristics of patients developing liver dysfunction after OAGB

Study	*n*	Age	Sex	Initial *BMI*	Liver biopsy	ALT (U/L)	AST (U/L)	GGT (U/L)	ALP (U/L)	Total bilirubin (mg/dL)	Albumin (g/dL)
Hussain et al. (2019)	2	*Mean*, 44	F	50	NA	NA	NA	NA	NA	NA	NA
			F	52	NA	NA	NA	NA	NA	NA	NA
Motamedi et al. (2017)	1	37	F	55.7	NA	23	16	NA	64	0.7	4.1
Al-Garzaie et al. (2022)	1	52	F	45	NA	NA	NA	NA	NA	NA	NA
Elgeidie et al. (2020)	2	62	F	41.3	NA	NA	NA	NA	NA	NA	NA
		56	M	63.7	NA	NA	NA	NA	NA	NA	NA
Sotelo et al. (2023)	1	20	F	43.1	NA	NA	NA	NA	NA	NA	NA
Kruschitz et al. (2016)	23	Mean, 43.8	NA	Mean, 45.3	NA	Mean, 30	Mean, 25	NA	Mean, 80	NA	NA
Hussain et al. (2018)	2	N/A	N/A	N/A	NA	NA	NA	NA	NA	NA	NA
Khalaj et al. (2019)	2	57	F	42.8	NA	31	28	NA	NA	NA	52
		37	F	44	NA	23	16	NA	NA	NA	41
Ahuja et al. (2018)	1	NA	NA	N/A	NA	NA	NA	NA	NA	NA	NA
Haddad et al. (2020)	1	42	F	57.5	NA	NA	NA	NA	NA	NA	NA
Eilenberg et al. (2017)	5	Mean, 40	F	42.8	Resolved NASH, broad fibrosis	NA	NA	NA	NA	NA	NA
			F	42.9	NA						
			F	40.8	NA	NA	NA	NA	NA	NA	NA
			F	57.6	NA	NA	NA	NA	NA	NA	NA
			M	64	NA	NA	NA	NA	NA	NA	NA
Kermansaravia et al. (2016)	1	29	F	55.7		22	128	NA	342	7.7	2.3
Motamedi et al. (2017)	1	57	F	42.8	NAFLD activity score 2/8	38	25	33	NA	1	5.2
Spivak et al. (2017)	47	Mean, 45	NA	Mean, 42.1	NA	Within normal levels	Within normal levels	Within normal levels	Within normal levels	NA	NA
Van Golen et al. (2022)	1	37	F	45.5	NA	NA	NA	NA	NA	NA	NA

**Table 3:** Postoperative characteristics of patients developing liver dysfunction after OAGB

Study	*n*	BPL length (cm)	Postoperative *BMI* (kg/m^2^)	%Excess weight loss at 1 year (%)	Liver biopsy	ALT (U/L)	AST (U/L)	GGT (U/L)	ALP (U/L)	Total bilirubin (mg/dL)	Albumin (g/dL)	Onset of liver dysfunction (month)
Hussain et al. (2019	2	300	NA	NA	NA	NA	NA	NA	NA	NA	NA	NA
		350	NA	NA	NA	NA	NA	NA	NA	NA	NA	NA
Motamedi et al. (2017	1	200	NA	104.1	Non-specific inflammation in portal spaces and fatty change	60	43	NA	395	1.1	1.8	12
Al-Garzaie et al. (2022	1	200	18	NA	Non-alcoholic steatohepatitis	NA	NA	NA	NA	NA	NA	6
Elgeidie et al. (2020	2	150	29	NA	NA	NA	NA	NA	NA	NA	2.2	8
		180	42	NA	NA	NA	NA	NA	NA	NA	1.9	10
Sotelo et al. (2023	1	200	15.11	NA	NA	29	84	110	153	6.54	2.59	4
Kruschitz et al. (2016	23	200	NA	127%	NA	35	29	NA	90	NA	NA	NA
Hussain et al. (2018	2	300	NA	NA	NA	NA	NA	NA	NA	NA	52	NA
Khalaj et al. (2019	2	200	NA	97.7	NA	54	33	NA	NA	NA	33	11.7
		200	NA	104.1	NA	64	83	NA	NA	NA	18	11.8
Ahuja et al. (2018	1	250	NA	NA	NA	NA	NA	NA	NA	NA	NA	3
Haddad et al. (2020	1	430	28	NA	NA	NA	NA	NA	NA	1.8	1.4	9
Eilenberg et al. (2017	5	175	18.4	125.4	Cirrhosis, steatosis	NA	NA	NA	NA	NA	NA	5
		370	20.8	123.7	Cirrhosis	NA	NA	NA	NA	NA	NA	12
		265	21.7	120.7	Cirrhosis	NA	NA	NA	NA	NA	NA	35
		200	22.4	107.8	Steatosis	NA	NA	NA	NA	NA	NA	36
		85	30.5	85.9	Steatosis	NA	NA	NA	NA	NA	NA	20
Kermansaravia et al. (2016	1	200	NA	NA	Steatohepatitis	22	128	NA	342	16.8	3.4	8
Motamedi et al. (2017	1	200			Prominent ballooning, steatosis, and neutrophilic satellitosis	38	33	NA	82	1.5	2.5	8
Spivak et al. (2017	47	200	NA	NA	NA	Mean, 64.8	Mean, 58.2	Mean, 97.3	Mean, 144.5	NA	NA	12
Van Golen et al. (2022	1	180	29	NA	Periportal and lobular hepatitis	428	723	NA	NA	247	24	7

### Prevalence of Liver Dysfunction in Patients Post-OAGB

**Fig. 2:**
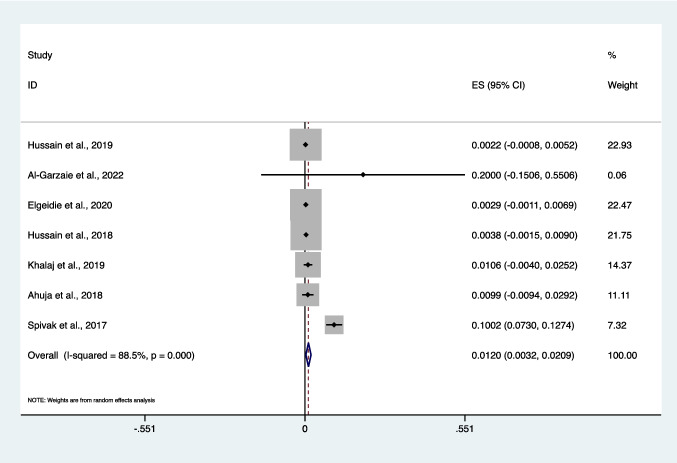
Forest plot showing the prevalence of liver dysfunction following one-anastomosis gastric bypass

Seven studies were included in the meta-analysis [7, 19, 20, 23, 26, 27, 30, 34]. The pooled estimate of the prevalence of liver dysfunction from these seven studies was 1.2% (95% *CI* 0.3–2.1%), with significant heterogeneity between the studies (*I*^2^ = 88.5%, *P* < 0.001) (Fig. 2).

### Subgroup Analysis

**Fig. 3:**
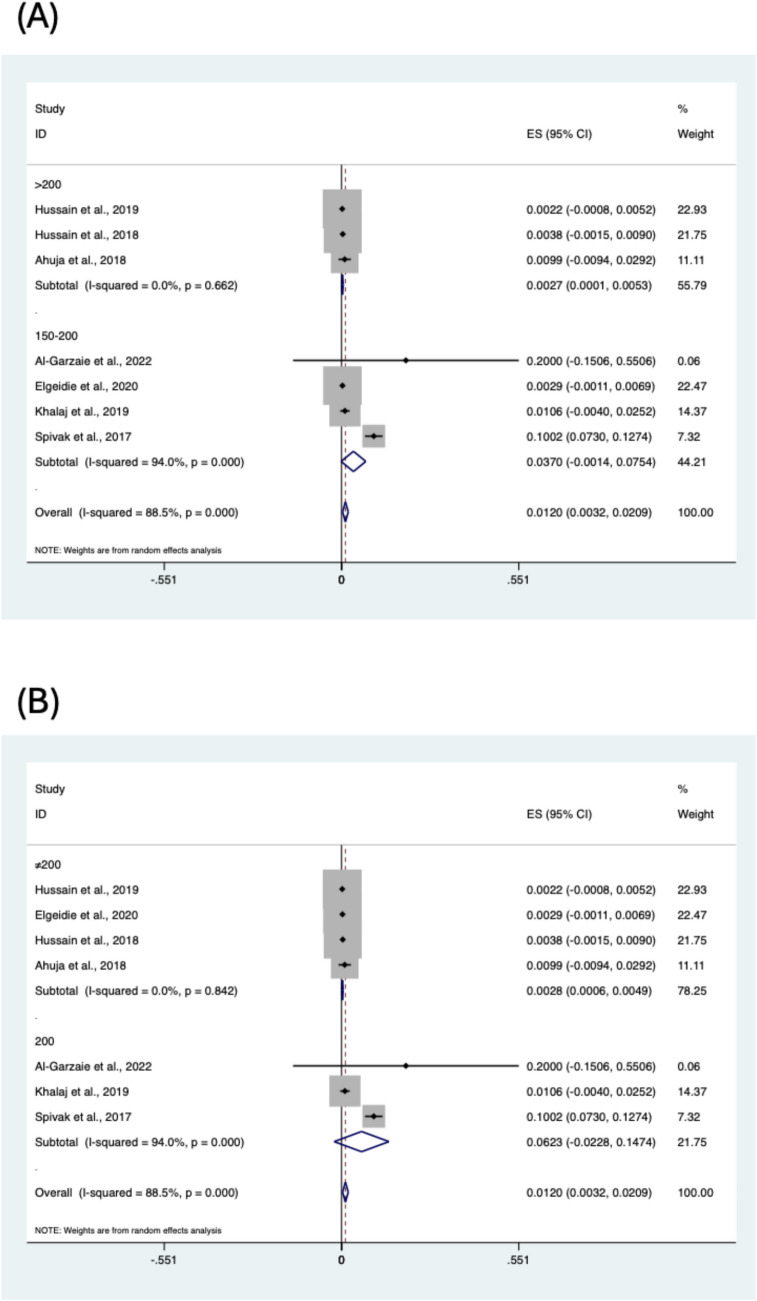
Subgroup analysis of liver dysfunction following one-anastomosis gastric bypass based on BPL length

**Fig. 4:**
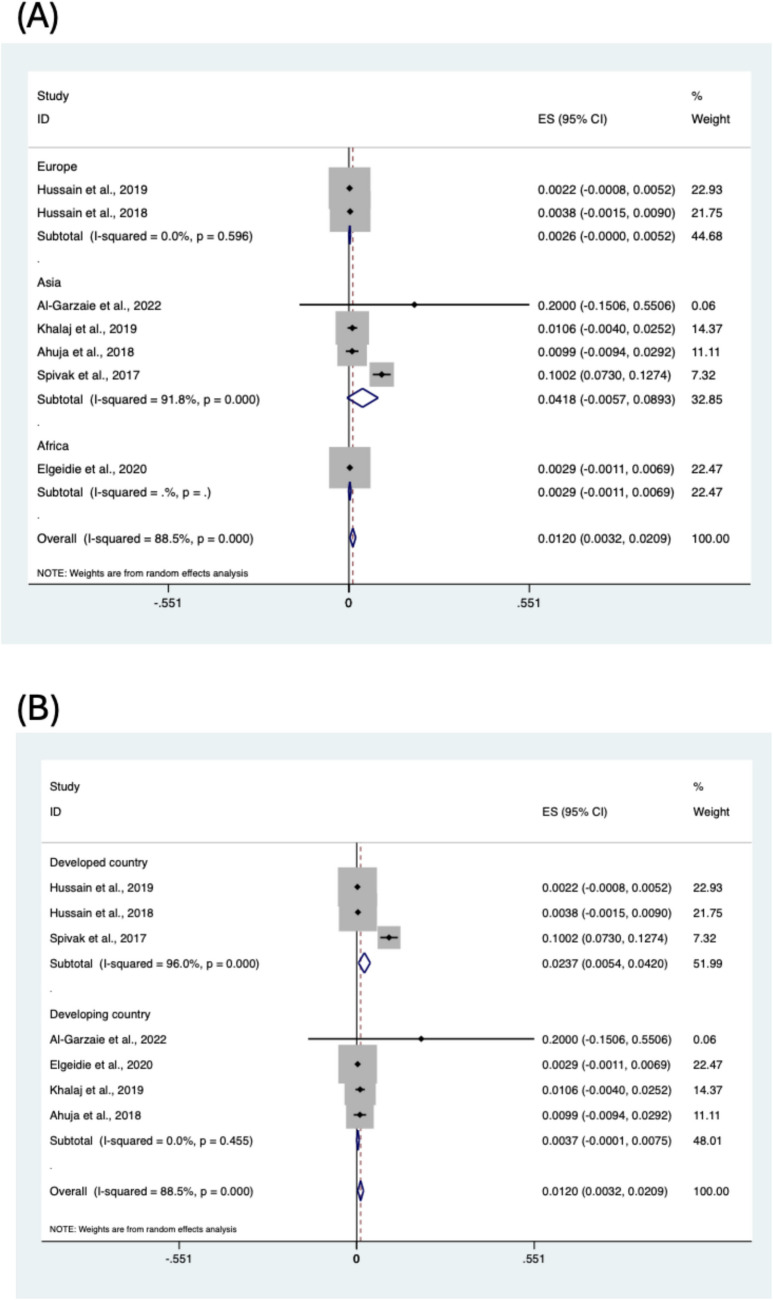
Subgroup analysis of liver dysfunction following one-anastomosis gastric bypass based on regions of included studies

Subgroup analyses were conducted to identify potential contributors to the high heterogeneity observed in the pooled prevalence estimate. Seven studies were included in the subgroup analysis [19, 20, 22, 23, 26, 27, 30]. Analyses were conducted by stratifying the data based on BPL length (150–200 cm vs. > 200 cm), BPL length (= 200 cm vs. ≠ 200) and regions of included studies (Figs. 3 and 4). However, the subgroup analysis could not find the potential contributors to heterogeneity. The prevalence of liver dysfunction was 0.37% in the 150–200 cm group and 0.27% in the > 200 cm group.

### Sensitivity Analysis

**Fig. 5:**
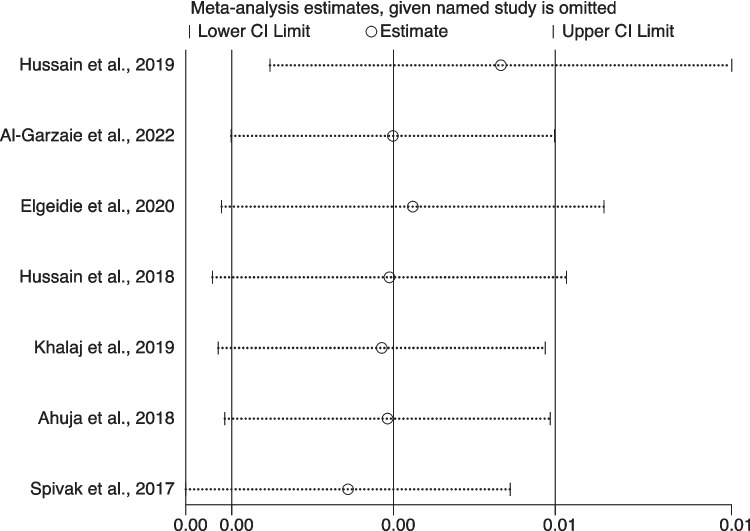
Sensitivity analysis of the meta-analysis

Sensitivity analysis was performed by omitting one study at a time to assess the robustness of our results. We found that the pooled result was not significantly affected by the exclusion of any single study (Fig. 5). This indicated that the results of this meta-analysis were statistically robust.

### Assessment of Publication Bias

**Fig. 6:**
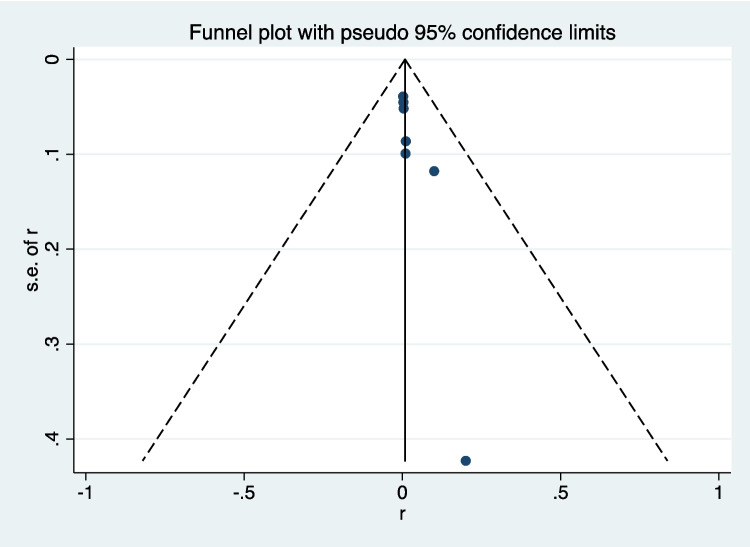
Funnel plot

**Fig. 7:**
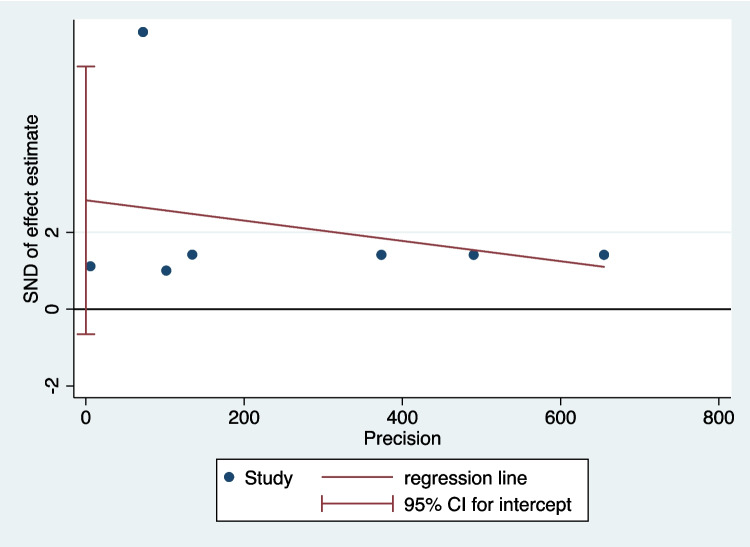
Egger’s funnel plot

The publication bias in the included studies was assessed by visual inspection of funnel plot (Fig. 6). The publication bias was then further assessed by Egger’s test. *P*-value of Egger’s test was 0.091. The results indicated no evidence of publication bias (Fig. 7).

## Discussion

This systematic review and meta-analysis represents the first comprehensive evaluation of liver dysfunction following OAGB, revealing a pooled prevalence of 1.2% (95% *CI* 0.3–2.1%) amongst 2944 patients across seven studies. Although this prevalence appears relatively low, the clinical ramifications as profound as liver dysfunction post-OAGB can progress to life-threatening complications including liver failure, transplantation or death [8, 21, 29, 31]. Our findings provide a foundation for understanding this serious complication and highlight the critical need for systematic postoperative liver function monitoring in OAGB patients. The 91 patients who developed liver dysfunction in our analysis presented with symptoms at a median of 12 months post-surgery (range 3–36 months), with postoperative *BMI*, ranging from 15.11 to 42 kg/m^2^ and median *BMI* of 25 kg/m^2^. This temporal pattern suggests that liver dysfunction typically manifests during the rapid weight loss phase, emphasizing the importance of vigilant monitoring during this critical period.

An important finding from our meta-analysis challenges the long-standing surgical principle that greater distalization increases the risk of hepatic failure. Traditional surgical doctrine suggests that longer biliopancreatic limbs should correlate with higher rates of liver dysfunction due to increased malabsorption. However, our subgroup analyses failed to demonstrate significant differences in liver dysfunction prevalence between different BPL length groups. Specifically, we found that 90% of patients who developed liver dysfunction had BPL lengths of 150–200 cm (prevalence 0.37%), whilst only 9% had BPL lengths > 200 cm (prevalence 0.27%). This unexpected finding warrants careful consideration of several possibilities. Firstly, the limited number of studies and patients with longer limb lengths may have resulted in insufficient statistical power to detect meaningful differences. Secondly, factors beyond limb length-such as individual patient characteristics, genetic predisposition, preoperative liver status or surgical technique variations—may be more important determinants of post-OAGB liver dysfunction than previously recognised [7, 26, 27]. Thirdly, the heterogeneity in limb length measurements and inconsistent reporting across studies may have obscured true associations.

In Roux-en-Y gastric bypass, emerging evidence suggests that the total alimentary limb length, defined as the sum of the common channel and Roux limb, is a more reliable predictor of malnutrition than BPL length alone [35]. This raises the possibility that the observed heterogeneity in our subgroup analyses may be attributable to the lack of standardised reporting of total alimentary limb length across studies. Specifically, patients in the short vs. long BPL groups may have had similar total alimentary limb lengths, thereby obscuring any true association between BPL length and the risk of liver dysfunction or malnutrition. This limitation highlights the need for future studies to report total limb configurations comprehensively to allow more meaningful comparisons. Our inability to identify limb length as a predictor suggests that current risk stratification models for post-OAGB liver dysfunction require fundamental reassessment, with important implications for surgical planning.

Our subgroup analyses attempted to identify potential contributors to the observed heterogeneity and lack of association between biliopancreatic limb length and liver dysfunction. We conducted analyses stratified by BPL length (150–200 cm vs. > 200 cm), BPL length (= 200 cm vs. ≠ 200 cm), and geographical regions of included studies, but none successfully explained the heterogeneity. The limited and inconsistent reporting of patient characteristics across studies severely constrained our ability to explore other potential risk factors. Most studies provided insufficient detail regarding preoperative liver status, with only a few reporting specific conditions such as fatty liver, steatosis or baseline liver function parameters [7, 8, 26]. Patient demographics varied considerably, with median ages ranging from 29 to 62 years and initial *BMI* from 41.3 to 63.7 kg/m^2^, but inconsistent reporting prevented meaningful subgroup analysis of these variables (Table 2). Additionally, variations in surgical technique, postoperative nutritional protocols, and follow-up monitoring practices were poorly documented across studies, representing unmeasured confounders that may influence liver dysfunction development. The predominantly retrospective nature of included studies [7, 19, 20, 23, 27, 30] further limited the availability of standardised baseline assessments that would enable comprehensive risk factor analysis. This highlights a critical need for future prospective studies with comprehensive baseline characterization and standardized outcome reporting to identify patient-specific and procedural risk factors for post-OAGB liver dysfunction.

These findings must be considered alongside recent long-term cohort data that raises additional concerns about bariatric surgery’s relationship with liver disease progression. Holmberg et al.’s four-country cohort study of 654,409 participants with obesity, including 86,356 who underwent bariatric surgery, demonstrated that bariatric surgery was associated with increased incidence (*HR* 1.23, 95% *CI* 1.11–1.37) and mortality (*HR* 1.93, 95% *CI* 1.50–2.48) of end-stage liver disease during a median follow-up of 7.3 years [36]. When analyses were restricted to gastric bypass procedures specifically, similar associations were observed for both incidence (*HR* 1.26, 95% *CI* 1.11–1.43) and mortality (*HR* 2.20, 95% *CI* 1.72–2.82) of end-stage liver disease. The study found that the incidence of end-stage liver disease decreased between 1 and 5 years after bariatric surgery but increased thereafter, suggesting a biphasic pattern that may not be captured in shorter follow-up studies. This apparent contradiction between short-term benefits and potential long-term risks highlights the complexity of bariatric surgery’s effects on liver health and emphasizes the need for extended surveillance protocols, particularly in patients with existing metabolic liver disease or other hepatic risk factors.

Multiple factors have been postulated to contribute to the pathogenesis of liver dysfunction post-OAGB. Malabsorption, especially amino acid deficiency, appears central to the pathophysiology, disrupting apolipoprotein B-100 synthesis, increasing free fatty acid mobilization and promoting hepatic fat accumulation [37]. Rapid and severe weight loss accelerates fat breakdown, leading to the release of inflammatory mediators and promoting lipotoxicity, which exacerbates the above process [38]. Additionally, anatomical changes post-OAGB predispose patients to small intestinal bacterial overgrowth (SIBO). Kaniel et al. conducted a prospective pilot study on 32 patients who underwent primary OAGB [39]. They found over one-third developed SIBO at 6 months following OAGB [39]. SIBO alters intestinal barrier function and increases intestinal permeability [40, 41]. Bacterial translocation to the portal venous system might directly induce hepatotoxicity, whilst SIBO increases bile deconjugation, further promoting hepatotoxicity [42, 43]. The complexity of these mechanisms suggests that effective prevention strategies must be multifaceted, addressing nutritional supplementation, weight loss velocity and potential SIBO prevention. However, the relative contribution of each mechanism remains unclear, necessitating further mechanistic research to guide-targeted interventions.

The clinical implications of these findings are immediate and significant for OAGB practice. The 1.2% prevalence rate, whilst seemingly modest, represents a clinically significant complication requiring systematic monitoring protocols. Currently, no studies in our analysis recommended specific frequencies for liver function monitoring, representing a critical gap in clinical guidance. Based on our findings, regular postoperative liver function monitoring is recommended, particularly during the first year when most complications manifest. Patients with preoperative liver pathology, severe obesity, insulin resistance or rapid weight loss patterns require especially vigilant surveillance [8]. We recommend regular follow-up and supplementation to prevent nutritional deficiencies, particularly in patients with prior severe obesity, insulin resistance, liver pathologies or rapid weight loss. The development of standardized monitoring protocols and nutritional supplementation guidelines represents an urgent clinical need.

Several limitations must be acknowledged. The substantial heterogeneity (*I*^2^ = 88.5%) across studies potentially stems from differences in study designs, patient populations or definitions of liver dysfunction. Most importantly, the sparse and inconsistent reporting of preoperative liver status is a significant shortcoming, as the majority of included studies did not indicate whether patients exhibited signs of chronic liver disease preoperatively, a limitation we acknowledge in the present study. The predominance of retrospective designs and the relatively small number of eligible studies further reduce the generalizability of the findings. Additionally, the lack of standardized definitions for liver dysfunction and inconsistent reporting of patient characteristics further complicates the interpretation of findings.

Unlike previous studies that primarily reported isolated cases or small cohorts of liver dysfunction following OAGB, this review provides a pooled estimate of prevalence, offering a broader perspective. Consistent with prior findings, this study reaffirms the potential severity of liver complications post-OAGB, aligning with the clinical experiences reported by Kruschitz et al. [8] and Eilenberg et al. [9]. However, the current review highlights the scarcity of large-scale, prospective studies on this topic [7, 19] and successfully addresses the research question by summarizing the current evidence on the prevalence and characteristics of liver dysfunction post-OAGB. Despite the limitations, the findings contribute valuable insights into the risk profile of OAGB, particularly regarding its impact on liver health, confirming the hypothesis that OAGB is associated with a measurable prevalence of liver dysfunction. Future research should focus on conducting large-scale, prospective studies to better elucidate the mechanisms, risk factors and long-term outcomes of liver dysfunction in OAGB patients. Standardized criteria for diagnosing and reporting liver dysfunction would enhance the comparability and reliability of future studies, whilst extended follow-up studies are essential to understand the full spectrum of OAGB’s hepatic effects given the concerning long-term liver disease risks suggested by recent cohort data [36].

## Conclusion

This systematic review and meta-analysis provide the first pooled prevalence estimate of liver dysfunction following OAGB, demonstrating a low but significant risk that warrants clinical attention. Importantly, our findings challenge traditional assumptions about the relationship between biliopancreatic limb length and liver dysfunction risk, suggesting that current risk stratification approaches may be inadequate. The findings underscore the importance of regular postoperative liver function monitoring in OAGB patients, potentially guiding clinical decision-making and patient counselling regarding the risks of this procedure. Despite its contributions, the review’s findings are limited by the heterogeneity and methodological weaknesses of the included studies, highlighting caution in extrapolating the results and emphasizing the necessity for systematic liver function monitoring, comprehensive preoperative assessment and the development of evidence-based prevention strategies to minimize this serious complication.

## Supplementary Information

Below is the link to the electronic supplementary material.

ESM 1 (DOCX 23.7 KB)

## Data Availability

The data supporting the findings of this study are available from the corresponding author upon reasonable request.
