# Supplementary material for: Prevalence of Liver Dysfunction After One-Anastomosis Gastric Bypass: A Systematic Review and Single-Arm Meta-analysis
Source: Obes Surg. 2025 Sep 9;35(10):4309–22. doi: 10.1007/s11695-025-08219-3 (PMC12540554; doi:10.1007/s11695-025-08219-3)
Supplement: Supplementary file 1 — (DOCX 23.7 KB) [file 11695_2025_8219_MOESM1_ESM.docx]

Supplementary file

Table S1. Search strategy

MEDLINE

| Search | Result |
| --- | --- |
| Ovid MEDLINE(R) ALL <1946 to June 27, 2024>  1 mini gastric bypass.mp. 363  2 one anastomosis gastric bypass.mp. 783  3 omega loop gastric bypass.mp. 66  4 single anastomosis gastric bypass.mp. 76  5 1 or 2 or 3 or 4 1072  6 limit 5 to (english language and full text and humans) 73 | 73 |

Embase

| Search | Result |
| --- | --- |
| #2. 'one anastomosis gastric bypass'/exp OR 'one anastomosis gastric bypass' OR 'omega loop gastric bypass':ab,ti OR 'mini gastric bypass':ab,ti OR 'single anastomosis gastric bypass':ab,ti | 2,305 |

PubMed

| Search | Result |
| --- | --- |
| ("mini gastric bypass"[Title/Abstract] OR "one anastomosis gastric bypass"[Title/Abstract] OR "omega loop gastric bypass"[Title/Abstract] OR "single anastomosis gastric bypass"[Title/Abstract]) AND ((fft[Filter]) AND (humans[Filter]) AND (english[Filter])) 845 | 845 |

Table S2. JBI Critical Appraisal Checklist for Case Reports

| JBI checklist | Were patient’s demographic characteristics clearly described? | Was the patient’s history clearly described and presented as a timeline? | Was the current clinical condition of the patient on presentation clearly described? | Were diagnostic tests or assessment methods and the results clearly described? | Were the intervention(s) or treatment procedure(s) clearly described? | Was the post-intervention clinical condition clearly described? | Were adverse events (harms) or unanticipated events identified and described? | Does the case report provide takeaway lessons? |
| --- | --- | --- | --- | --- | --- | --- | --- | --- |
| Motamedi et al., 2017 | Yes | Yes | Yes | Yes | Yes | Yes | Yes | Yes |
| Al-Garzaie et al., 2022 | Yes | Yes | Yes | No | Yes | Yes | Yes | Yes |
| Sotelo et al., 2023 | Yes | Yes | Yes | No | Yes | Yes | Yes | Yes |
| Khalaj et al., 2019 | Yes | Yes | Yes | Yes | Yes | Yes | Yes | Yes |
| Haddad et al., 2020 | Yes | Yes | Yes | No | Yes | Yes | Yes | Yes |
| Eilenberg et al., 2017 | Yes | Yes | Yes | Yes | Yes | Yes | Yes | Yes |
| Kermansaravia et al., 2016 | Yes | Yes | Yes | Yes | Yes | Yes | Yes | Yes |
| Motamedi et al., 2017 | Yes | Yes | Yes | Yes | Yes | Yes | Yes | Yes |
| Van Golen et al., 2022 | Yes | Yes | Yes | Yes | Yes | Yes | Yes | Yes |

Table S3. JBI Critical Appraisal Checklist for Cohort Studies

| JBI checklist | Were the two groups similar and recruited from the same population? | Were the exposures measured similarly to assign people? | To both exposed and unexposed groups? | Was the exposure measured in a valid and reliable way? | Were confounding factors identified? | Were strategies to deal with confounding factors stated? | Were the groups/participants free of the outcome at the start of the study? | Were the outcomes measured in a valid and reliable way? | Was the follow up time reported and sufficient to be long enough for outcomes to occur? | Was follow up complete, and if not, were the reasons to loss to follow up described and explored? | Were strategies to address incomplete follow up utilised? | Was appropriate statistical analysis used? |
| --- | --- | --- | --- | --- | --- | --- | --- | --- | --- | --- | --- | --- |
| Hussain et al., 2019 | No | No | Yes | No | Yes | Yes | Yes | Yes | Yes | Yes | Yes | Yes |
| Elgeidie et al., 2020 | Yes | Yes | Yes | No | Yes | Yes | Yes | Yes | Yes | Yes | Yes | Yes |
| Kruschitz et al., 2016 | No | No | No | No | Yes | No | Yes | Yes | Yes | Yes | Yes | Yes |
| Hussain et al., 2018 | No | No | No | No | Yes | Yes | Yes | Yes | Yes | Yes | Yes | Yes |
| Ahuja et al., 2018 | Yes | Yes | Yes | Yes | Yes | Yes | Yes | Yes | Yes | Yes | Yes | Yes |
| Spivak et al., 2017 | Yes | Yes | Yes | Yes | Yes | Yes | Yes | Yes | Yes | Yes | Yes | Yes |

Table S4. Cumulative Percentage of Liver Dysfunction by BPL Length Group

| BLP Group | Total Patients | Liver Dysfunction | Cumulative Percentage of Liver Dysfunction |
| --- | --- | --- | --- |
| <150 cm | 387 | 1 | 1% |
| 150-200 cm | 2532 | 82 | 90% |
| >200 cm | 24 | 8 | 9% |
